# Energy Sensing Pathways in Aging and Chronic Lung Disease

**DOI:** 10.1093/geroni/igaa057.2700

**Published:** 2020-12-16

**Authors:** Victor Thannickal

**Affiliations:** University of Alabama, Birmingham, Alabama, United States

## Abstract

The cause-effect relationships between the various “hallmarks of aging” and chronic lung disease are not well understood. We have determined overlapping pathways involving deregulated nutrient sensing, mitochondrial dysfunction, and cellular senescence that may contribute to the evolution of chronic lung disease. In particular, I will discuss alterations in energy/metabolic sensing pathways and mitochondrial dysfunction as pathobiological mechanisms that may explain the age-related increased susceptibility to the development and progression of idiopathic pulmonary fibrosis (IPF), a disease of pulmonary aging. I will then broaden the discussion to include the potential role of these biologic alterations in other chronic lung disease which burden older adults.

